# Crisis-related stimuli do not increase the emotional attentional blink in a general university student population

**DOI:** 10.1186/s41235-023-00525-7

**Published:** 2024-01-08

**Authors:** Lindsay A. Santacroce, Benjamin J. Tamber-Rosenau

**Affiliations:** 1 Department of Psychology, University of Houston, Fred J. Heyne Building, Room 126, 3695 Cullen Blvd, Houston, TX 77204 USA; 2 Department of Psychology, Toronto Metropolitan University, Jorgenson Hall, 9th Floor, 380 Victoria Street, Toronto, ON M5B 2K3 Canada

**Keywords:** Emotional attentional blink, Emotion-induced blindness, Emotional capture, Natural disaster, Pandemic

## Abstract

Crises such as natural disasters or pandemics negatively impact the mental health of the affected community, increasing rates of depression, anxiety, or stress. It has been proposed that this stems in part from crisis-related stimuli triggering negative reactions that interrupt daily life. Given the frequency and prominence of crisis events, it is crucial to understand when crisis-related stimuli involuntarily capture attention and trigger increased stress and distraction from obligations. The emotional attentional blink (EAB) paradigm—in which emotional distractors hinder report of subsequent targets in streams of rapidly displayed stimuli—allows examination of such attentional capture in a rapidly changing dynamic environment. EABs are typically observed with generally disturbing stimuli, but stimuli related to personal traumas yield similar or greater effects, indicating strong attentional capture by stimuli related to individual trauma history. The current study investigated whether a similar comparable or increased crisis-related EAB exists within a community affected by large-scale crisis. Specifically, effects of conventional emotional distractors and distractors related to recent crises were compared using EABs in university students without a mental health diagnosis. Experiment 1 used images related to Hurricane Harvey, evaluating a crisis 4 years prior to data collection. Experiment 2 used words related to the COVID pandemic, evaluating an ongoing crisis at the time of data collection. In both experiments, the conventional EAB distractors yielded strong EABs, while the crisis-related distractors yielded absent or weak EABs in the same participants. This suggests that crisis-related stimuli do not have special potency for capturing attention in the general university student population. More generally, crises affecting communities do not necessarily yield widespread, strong reactivity to crisis-related stimuli.

**Supplementary Information:**

The online version contains supplementary material available at 10.1186/s41235-023-00525-7.

## Introduction

Whether it be a natural disaster, global pandemic, war, or another similar crisis, distressing events affect entire communities and can lead to widespread fear, stress, depression, and anxiety related to the event (Makwana, 2019). With the frequency and severity of such crises rapidly increasing as a result of, among other things, climate change, the exhaustion of non-renewable resources, and the fragile global economy, it is important to understand how these events affect the day-to-day lives of the general population. Stress from crises not only directly impact mental states, but may also change the relative salience of crisis-related stimuli, which can act as cues that trigger unintentional emotional responses. Such emotional responses can influence the control of selective attention, which is a constantly used component of nearly all perception and cognition and plays a large role in day-to-day functioning.

Humans are constantly being bombarded with perceptual information, and thus it is crucial to allocate attention to stimuli consistent with current goals while ignoring distracting information. However, highly salient stimuli can capture attention even in spite of current goals, hindering one’s ability to complete volitional tasks. One potent source of salience that can lead to attentional capture is emotional salience, i.e., strong emotional valence and/or arousal. Emotionally driven capture often occurs in generally unpleasant situations, such as the natural tendency to “rubberneck” attention toward a car crash, which can interfere with attention allocated toward driving and cause a second crash. Similarly, stimuli related to personally relevant stressful events can exacerbate attentional capture because such events can lead to subsequent intrusive thoughts or memories, and can instantly take priority over current goals, even if the triggering stimuli would be seen as neutral to others who did not experience the stressful event (Krans et al., 2012).

In the laboratory, extensive research has examined the effects of stressful events, mostly focusing on relatively severe cases, such as in individuals who develop post-traumatic stress disorder (PTSD), clinical depression, or clinical anxiety. Compared to control participants, participants with and without clinically diagnosed PTSD who were recently exposed to trauma display accelerated heart rates when they are subsequently shown stimuli related to their personal trauma; moreover, they fixate on trauma-related stimuli longer than they do disturbing stimuli that are not tied to their recent experience (Elsesser et al., 2004). It has also been shown that combat veterans tend to rate combat-related words as being more arousing than control participants, and that combat veterans diagnosed with PTSD rate the same words as being even more arousing (Todd et al., 2015).

Stimuli related to stressful events have also been shown to capture attention during computerized tasks, possibly by evoking intrusive memories or otherwise making it more difficult to inhibit the distracting items and complete the task at hand. A common demonstration of this is seen in the emotional Stroop task (Williams et al., 1996), in which participants are to report the font color of words that are either neutral or emotional. Research has consistently shown that participants respond to the color of the emotional words more slowly than the neutral words, indicating a general difficulty in inhibiting distraction by emotional stimuli (Williams et al., 1996). However, the effect of emotion is magnified by trauma exposure: participants exposed to trauma (e.g., combat, assault) were negatively affected by words relating to their trauma more so than controls (Cisler et al., 2011). Even healthy participants who had not been exposed to a traumatic event have demonstrated emotional Stroop interference when the words were related to a personal emotional or stressful event (Wingenfeld et al., 2006), suggesting that even non-traumatic events in non-clinical samples can elicit intrusive memories that interfere with attention and inhibitory control.

The emotional Stroop task is appropriate to observe emotional interference with single events (presentation of colored text), but it may not be ideal for capturing variability in attentional interference experienced during real-world events, which involve rapidly changing dynamic information. Another common paradigm used to study attentional control addresses this constraint by embedding one or more targets in a rapidly changing stream of filler stimuli, known as the rapid serial visual presentation (RSVP) task. RSVP tasks are often used to study a phenomenon known as the attentional blink (AB), in which the processing of a first target (T1) diminishes the ability to successfully report the second target (T2) when they are separated by a short temporal lag (Broadbent & Broadbent, 1987; Raymond et al., 1992). Because increasing the lag between T1 and T2 rapidly attenuates the blink, this paradigm can be used to examine the dynamic limits of attentional control across time. Crucially, previous AB research manipulating the valence of T2 has shown that an emotional T2 can capture attention and attenuate the AB effect (e.g., Keil & Ihssen, 2004), which is more apparent in soldiers when T2 was combat-related, and even more so in soldiers with PTSD (Todd et al., 2015). Similarly, eliciting personal stressors using familiar emotional images can result in a “backwards blink,” or poorer T1 performance (Krans et al., 2012).

While the AB paradigm is suitable for examining emotional capture in dynamic settings, asking participants to attend to the emotional stimulus as one of the two to-be-reported targets fails to address emotional capture by *distracting* stimuli, which might more accurately reflect intrusions by unwanted thoughts. However, a closely related RSVP paradigm is used to study a phenomenon known as emotion-induced blindness (EIB), in which a single task-*irrelevant* emotional stimulus can capture attention and transiently interrupt detection or identification of a subsequent target (Most et al., 2005). Thus, the EIB creates a “blink” similar to that in the AB, but as a result of an emotional distractor rather than a T1. The EIB is also known as the emotional attentional blink (EAB), which is the term we use below.

Only limited research has examined how the EAB is modulated by stressful events, and this research has yielded mixed results. Specifically, an increased EAB effect has been observed in combat veterans with PTSD when the emotional distractor was combat-related, while the same was not true in combat veterans without PTSD or control participants (Olatunji et al., 2013). On the other hand, Olatunji et al. (2022) showed that combat-related emotional stimuli did not yield a stronger EAB compared to more typical EAB stimuli (i.e., disgust), even when the participants were combat-exposed veterans with or without PTSD. When a stressful “event” was elicited using a disturbing video clip in healthy participants, those who were most negatively affected by the event (reported more intrusive episodes) also experienced greater attentional capture in the EAB from stimuli related to that event (Verwoerd et al., 2009). Similarly, research has examined how other clinical measures relate to the EAB and have shown that lower levels of harm avoidance correlate with better inhibitory strategies to reduce the EAB effect (Most et al., 2005), higher trait anxiety yielded a prolonged EAB (Chen et al., 2020), greater negative affect correlated with an increased EAB effect that was mediated by persistent negative thought (Onie & Most, 2017), and generalized anxiety disorder resulted in a greater EAB effect (Olatunji et al., 2011).

The approaches reviewed above evaluate the effects of stressful emotionally laden stimuli on the EAB, which is beneficial for understanding how the presence of stressor-related distractor stimuli can interfere with attention toward current goals. However, many of these methods are less applicable to the general population because they rely on examining small subsets of the population (clinical samples of trauma-exposed participants) or using individual measures (levels of negative affect) to predict the typical EAB effect. Others artificially create a stressful event in attempts to mimic real crises, which raises concerns of generalizability to real-world stress. None of the extant approaches captures potential subclinical effects of widespread exposure to a shared stressful event among a large community of people. Given the growing prevalence of catastrophic events that affect regions (e.g., natural disasters) or the entire globe (e.g., pandemics; Marani et al., 2021), it is equally as important to understand how current and recent crises affect cognition in the general population as it is to understand the effects of these crises in the most-affected individuals. The present research aims to study the ability to disengage from task-irrelevant crisis-related stimuli using the EAB paradigm in a population that represents a diverse range of moderate to severe impacts from two crises—university students previously exposed to Hurricane Harvey and currently in the midst of the coronavirus disease 2019 (COVID) pandemic.

## Experiment 1

Hurricane Harvey devastated southeast Texas when it made landfall as a category 4 hurricane on August 25th, 2017, and is considered one of the most destructive hurricanes of all time (Blake & Zelinsky, 2018). Considering that the current study was conducted in Houston, Texas—perhaps the most heavily impacted city—this crisis was optimal for the present investigation because any individual in the greater Houston area during Harvey was negatively affected by the crisis, many still living in its wake to this day. Lingering visible impacts throughout the Houston area, including demolished buildings and homes, halted infrastructure construction projects, and approximately 11,000 people still awaiting emergency aid five years later (Watkins, 2022), serve as constant reminders of the tragedy and likely contribute to lasting mental health issues. Notably, University of Houston students are a population that may be less insulated from the effects of crisis compared to university students nationally: of the undergraduate population, 74% are considered a part of a minority race or ethnicity (CollegeSimply, 2023), 45% are first-generation college students (University of Houston, 2022), and 44% are considered low-income (CollegeSimply, 2023). Therefore, Experiment 1 examined the potential for EABs to be evoked by crisis-related images depicting the city of Houston immediately following Hurricane Harvey. Data for Experiment 1 were collected between August and November of 2021, or four years following the Hurricane, which allows for examination of lingering long-term stress resulting from a crisis event.

### Method

#### Participants

A total of 40 University of Houston students (36 females, 3 males, 1 non-binary; *M*_age_ = 21.28, SD_age_ = 4.02) participated in Experiment 1 for course credit through the university’s SONA system. Participants were at least 18 years of age, had normal or corrected vision, did not report color blindness, did not report regular or task-concurrent use of psychoactive drugs, and did not report having neurological disorders, brain injuries, or other diagnoses known to affect cognition. All participants reported that they were in Houston or in nearby areas at the time of Hurricane Harvey. Informed consent was gathered from all participants under a protocol approved by the University of Houston Institutional Review Board.

#### Sample size justification

A sample size of 40 participants was initially selected for Experiment 1 (and Experiment 2) because it is common in similar AB/EAB studies (Kennedy & Most, 2015; Santacroce et al., 2021). Because the analyses rely primarily on Bayesian hypothesis tests (see data analyses below for more information), the results are not biased toward or away from supporting the null or alternative hypothesis based upon sample size, and an inadequate sample size would yield Bayes factors of approximately 1. The initial sample yielded sufficient evidence for the key statistical tests, characterized by the Bayes factors (BF_inc_s) above 3 or less than 1/3 (Jeffreys, 1961; Wagenmakers et al., 2018), and so 40 participants were satisfactory for the current experiments.

#### Design and procedure

**Fig. 1:**
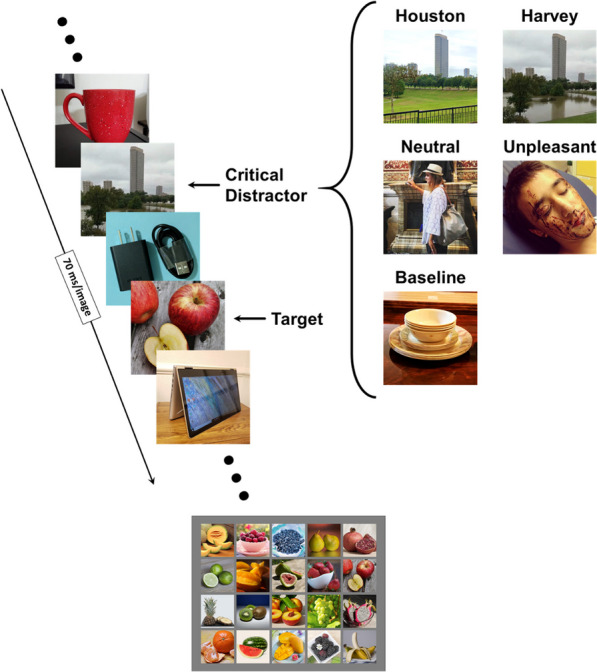
Visual representation of the RSVP task used in Experiment 1. Participants viewed a stream of filler images of objects presented at a rate of 70 ms per image with a fruit target image and a critical distractor preceding the target. Following each trial, participants used their mouse to select the fruit image they saw from an array of other fruit images. The right side of the figure shows examples of critical distractors from the Houston, Harvey, neutral, unpleasant, and baseline valence conditions. The unpleasant and neutral distractors presented in this figure are not part of the IAPS images that were used in the actual experiment because of constraints on the public dissemination of IAPS images. The IAPS images used in the neutral and unpleasant conditions depicted humans both close-up (such as the unpleasant image depicted here) and further away (such as the neutral image depicted here). The trial stream here depicts a Harvey lag 2 trial

To test if stress related to Hurricane Harvey yields an EAB, Experiment 1 used an established EAB RSVP paradigm (Santacroce et al., 2023) in which a series of images is presented, one at a time, in the same spatial location on a computer screen. Each trial was started by pressing the space key, which initiated a RSVP stream with 2–4 filler items, a critical distractor, 0 (lag 1), 1 (lag 2), 3 (lag 4), or 9 (lag 10) filler items, a previously defined target, and 3–14 additional fillers, for a total of 18 stimuli per RSVP stream. Each image was presented for 70 ms, which is consistent with a previous study using the same paradigm (Santacroce et al., 2023), and thus one RSVP stream lasted for a total of 1260 ms. The filler images contained common everyday objects. Targets were defined as images of fruit; thus, target selection required semantically processing all RSVP images. Critical distractors could be one of five valence categories: baseline (critical distractor replaced with a filler item), neutral, unpleasant, Houston, or Harvey (Fig. 1). Following each trial, participants were presented with an array of 20 fruit images and selected the fruit target they saw using their mouse.

Prior to beginning the experiment, participants provided consent and filled out a demographics survey on Qualtrics, completed a screen scale calibration program (see below), were given on-screen instructions, and carried out five practice trials. The practice trials contained simpler images (object-only photographs with solid white backgrounds), never contained a critical distractor, only displayed nine images per stream, and had only nine fruit images to choose from. The first practice trial was presented at a rate of 350 ms per image, and that rate decreased by 70 ms for each trial until it reached the experiment’s speed of 70 ms per image on the fifth practice trial. Participants were given feedback following each practice trial, while no feedback was presented after the practice period.

With the five valence categories (baseline, neutral, unpleasant, Houston, or Harvey) and four critical distractor to target lags (lags 1, 2, 4, and 10), Experiment 1 had a total of 20 conditions that each had 30 trials for a total of 600 trials. Each session, including consent, demographic collection, setup procedures, training trials, and the experiment trials, lasted approximately 1.5 h.

#### Apparatus

The task in Experiment 1 (and Experiment 2) was constructed using the PsychoPy experiment builder (Peirce et al., 2019) and was hosted on Pavlovia.org. Participants first provided consent, reported demographic information, and read the task instructions on a Qualtrics.com survey, accessed through a link on the University of Houston’s Sona System. Upon completion of the Qualtrics survey, participants were directed to the online experiment. To ensure they understood the task, participants were given detailed instructions on the first few screens of the experiment, and then completed the five practice trials before continuing to the real experiment.

Because the experiments in this study were conducted online, a number of measures were taken to combat issues that might accompany an online study. In order to keep stimulus size uniform across different participants’ monitors, participants also completed a credit card screen scale calibration (Morys-Carter, 2020) at the start of the experiment. Specifically, an image of a credit card was displayed on the screen and participants were instructed to hold up a credit card to the screen and adjust the image until it precisely matched the size of the credit card. The output from the credit card calibration was used to scale the task stimuli to these dimensions, regardless of the participants’ monitors. In order to control for the refresh rate of individual participants’ monitors and to avoid visible latency variations during the RSVP streams, the stimulus presentation rate was the number of frames nearest to the desired display time (here 70 ms), which was calculated individually for each participant. To combat setting variability and to maximize task performance, participants were instructed to complete the experiment while sitting up straight at a table in a secluded room, minimize distractions as much as possible (put away cell phone, do not listen to music, do not have the TV on, etc.), complete the experiment on a computer or laptop (no phones or tablets), close all other programs and Internet browser tabs, use the Google Chrome or Microsoft Edge browser, plug in laptops with battery saver off, and complete the experiment in one sitting (although they could take short breaks between trials as needed). Of note, prior online RSVP experiments conducted by the authors have used these control measures and have observed expected results (Santacroce et al., 2021, 2023), including two different EAB experiments (one of which was the paradigm used here in Experiment 1) that included direct replications of in-laboratory experiments and yielded nearly identical results (Santacroce et al., 2023).

#### Stimuli

All task images contained a clear central subject with naturalistic background scenes spanning the entire extent of the image. Twenty target images of recognizable fruits were collected from publicly available sources and contained both the whole fruit and a sliced fruit. Twenty-one filler images of common everyday objects (mug, purse, shoes, etc.) were hand-selected from publicly available sources to approximately match the colors and clarity of the fruit images. Eight object images and nine fruit images that were simpler on a plain white background were used for the practice trials. All images were approximately 11.68 cm × 11.68 cm (made uniform across participants’ monitors by the credit card screen scale calibration) and centered on the screen.

In the baseline valence condition, there was an additional object filler image in place of a critical distractor in order to serve as a true control condition. There were two emotional conditions in Experiment 1: the conventional EAB condition in which the critical distractors were commonly used unpleasant images of humans, and the stress-induced Harvey condition in which the critical distractors were images of Houston in the wake of Hurricane Harvey. Because the two emotional conditions used images from different categories than the surrounding RSVP stimuli (humans and cities vs. objects and fruits), and because the Harvey images showcased information personally relevant to the participants (where they lived), it was possible that a capture effect could be driven by these factors rather than emotion (Baker et al., 2021; Santacroce et al., 2023). Thus, each emotional condition had a matching neutral control condition using images from the same category. For the conventional unpleasant condition, the images of unpleasant humans were matched with a neutral condition with images of neutral humans. For the stress-induced Harvey condition, the images of Houston after Harvey were matched with images of Houston prior to Harvey.

The unpleasant critical distractor images and their neutral control images were collected from the International Affective Picture System (IAPS; Lang et al., 2008) database based on normative valence and arousal ratings using the Self-Assessment Manikin (Bradley & Lang, 1994) 9-point scale. The neutral distractors consisted of 72 images of humans in neutral settings and the unpleasant distractors consisted of 72 images of humans in unpleasant/gory settings. The critical distractor IAPS images were hand-selected and hand-cropped into squares to assure that the neutral images were truly neutral (arousal: mean = 3.79, SD = 0.62; valence: mean = 5.85, SD = 1.07), the unpleasant images were the most graphic (arousal: mean = 6.28, SD = 0.65; valence: mean = 2.16, SD = 0.63), and the cropping did not cut out the main subject of the images.

The Houston and Harvey images were collected from a Houston Chronicle article that showcased side-by-side images of locations in Houston, Texas, both before and immediately following Hurricane Harvey (Gordon, 2018). The Harvey images taken after Hurricane Harvey depicted mass flooding, heavily damaged homes, and citizen evacuations. The Houston images depicted the city of Houston on an average day taken in the exact same locations as the Harvey images with identical framing. There were a total of 48 matched pairs of images used for the Houston and Harvey valence categories. Crucially, the Harvey images were rated as being more negative, arousing, and crisis-related than the neutral Houston images by a separate set of participants (*n* = 47, *p*s < 0.001, BF_10_s ≥ 100.37; see Additional file 1).

#### Data analyses

Data were tabulated using custom MATLAB code and all subsequent analyses were completed in the JASP statistical program (JASP Team, 2018). Both Bayesian statistical tests (e.g., Bayesian ANOVA) and their frequentist equivalents (e.g., ANOVA) were conducted to remain consistent with a majority of previous EAB studies (that rely on frequentist hypothesis testing), while also providing a more complete picture of the results (Wagenmakers et al., 2022). The main analysis of interest was a 5 (valence: neutral, unpleasant, Houston, Harvey, baseline) × 4 (lag: 1, 2, 4, 10) within-subjects ANOVA to indicate a difference in the blinks caused by the different valence categories. A series of follow-up ANOVAs were conducted to detect possible EAB effects in each valence condition. First, individual one-way ANOVAs were conducted within each valence category to examine a possible blink effect, indicated by a main effect of lag, which is commonly used in AB studies, but has also been used in EAB paradigms (Santacroce et al., 2023). Second, valence × lag ANOVAs were conducted to compare each of the conditions to the baseline condition, and then, each emotional condition (unpleasant and Harvey) to their matched neutral counterpart condition (neutral and Houston, respectively), which are common ways to quantify an EAB effect. Finally, to directly compare the conventional EAB effect to the stress-induced EAB, a valence × lag ANOVA was conducted with the unpleasant and Harvey valence conditions.

Each frequentist test was accompanied by a Bayesian ANOVA calculated across matched models stripped of the effects (also known as Baws factor; Mathôt, 2017) to quantify support for or against including each potential main effect or interaction in the model. As introduced by Jeffreys (1961), BF_inc_s greater than 1 are interpreted as evidence for including the effect (support interpretation levels: 1–3 anecdotal, 3–10 moderate, 10–30 strong, 30–100 very strong, and > 100 extreme) and values less than 1 are interpreted as evidence against including the effect (support interpretation levels: 1/3–1 anecdotal, 1/10–1/3 moderate, 1/30–1/10 strong, 1/100–1/30 very strong, and < 1/100 extreme).

### Results and discussion

**Fig. 2:**
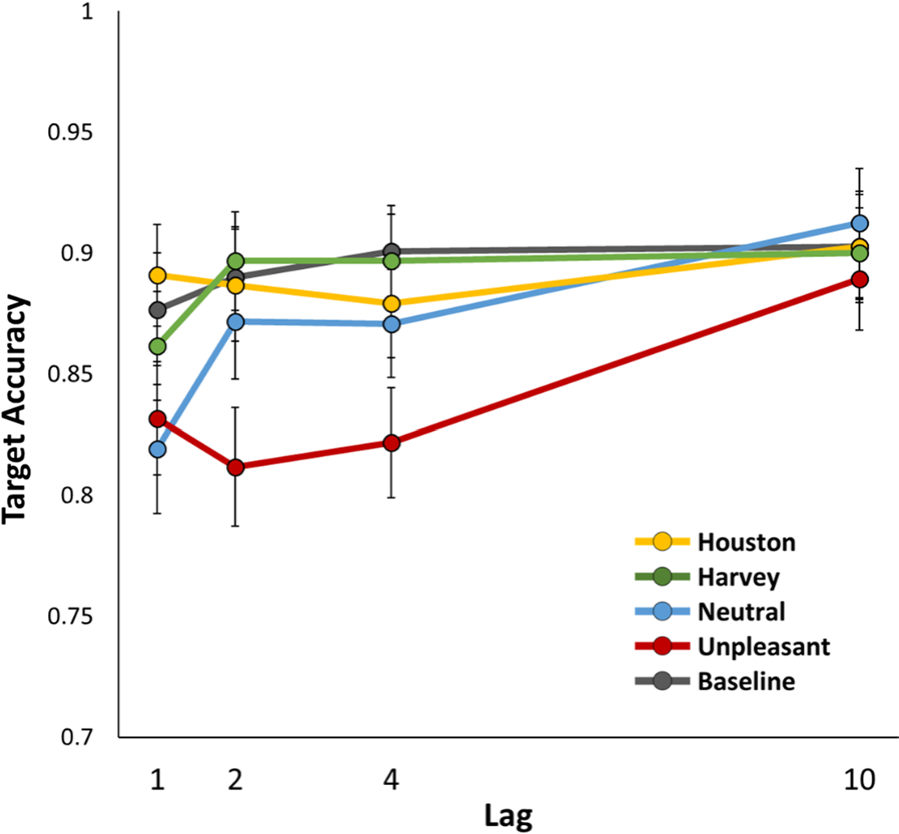
The results of Experiment 1. Error bars represent the standard error of the mean

See Table 1 for full outputs from each ANOVA in Experiment 1. Crucially, the initial 5 (valence: neutral, unpleasant, Houston, Harvey, baseline) × 4 (lag: 1, 2, 4, 10) within-subjects ANOVA yielded a valence × lag interaction (*p* < 0.001, *η*_*p*_^2^ = 0.10, BF_inc_ = 3.48 × 10^3^), indicating a difference in the “blinks” yielded by each valence category (Fig. 2). The follow-up analyses yielded a conventional (unpleasant) EAB effect, indicated by valence × lag interactions when the unpleasant condition was compared to the baseline condition (*p* = 0.004, *η*_*p*_^2^ = 0.11, BF_inc_ = 6.61) and the neutral condition (*p* = 0.003, *η*_*p*_^2^ = 0.11, BF_inc_ = 6.36), and by a simple main effect of lag in the unpleasant condition (*p* < 0.001, *η*_*p*_^2^ = 0.17, BF_inc_ = 406.96). On the other hand, the stress-induced (Harvey) blink was ambiguous: there was a main effect of lag in the one-way ANOVA on the Harvey condition (*p* = 0.006, *η*_*p*_^2^ = 0.10, BF_inc_ = 5.54), but there was not clear evidence of a valence × lag interaction when the Harvey condition was compared to the Houston condition (*p* = 0.036, *η*_*p*_^2^ = 0.07, BF_inc_ = 1.17) or the baseline condition (*p* = 0.644, *η*_*p*_^2^ = 0.01, BF_inc_ = 0.06). Regardless of the statistical test used to evaluate the presence of an EAB in each condition, the primary takeaway from these results is that, contrary to the initial hypothesis, the stress-induced EAB evoked by images of Hurricane Harvey was not comparable to or greater than the conventional EAB evoked by unpleasant images; instead, it was significantly smaller (*p* < 0.001, *η*_*p*_^2^ = 0.13, BF_inc_ = 23.90).

**Table 1:** Results for each analysis of variance ran in Experiment 1

Source	*df*^a^	*F*	*p*	*η*_*p*_^2^	*BF*_inc_^b^
All valence conditions × lag					
Lag	(3, 117)	16.01	< .001	0.29	1.22 × 10^9^
Valence	(2.81, 109.65)	22.63	< .001	0.37	3.53 × 10^14^
Valence × lag	(8.05, 109.65)	4.47	< .001	0.10	3.48 × 10^3^
Unpleasant versus baseline × lag					
Lag	(3, 117)	7.88	< .001	0.17	184.09
Valence	(1, 39)	42.47	< .001	0.52	6.91 × 10^8^
Valence × lag	(3, 39)	4.66	.004	0.11	6.61
Unpleasant versus neutral × lag					
Lag	(3, 117)	17.95	< .001	0.32	7.72 × 10^7^
Valence	(1, 39)	13.68	< .001	0.26	141.08
Valence × lag	(3, 39)	4.86	.003	0.11	6.36
Harvey versus baseline × lag					
Lag	(3, 117)	6.02	< .001	0.13	42.15
Valence	(1, 39)	0.46	.501	0.01	0.15
Valence × lag	(3, 39)	0.56	.644	0.01	0.06
Harvey versus Houston × lag					
Lag	(3, 117)	3.04	.032	0.07	0.65
Valence	(1, 39)	0.03	.858	8.37 × 10^−4^	0.13
Valence × lag	(3, 39)	2.94	.036	0.07	1.17
Unpleasant versus Harvey × lag					
Lag	(3, 117)	7.92	< .001	0.17	237.85
Valence	(1, 39)	37.38	< .001	0.49	2.26 × 10^7^
Valence × lag	(3, 39)	5.90	< .001	0.13	23.90
Simple main effects of lag					
Unpleasant	(3, 117)	8.23	< .001	0.17	406.96
Neutral	(3, 117)	18.25	< .001	0.32	1.09 × 10^7^
Harvey	(3, 117)	4.41	.006	0.10	5.54
Houston	(3, 117)	1.42	.242	0.04	0.17
Baseline	(3, 117)	2.10	.105	0.05	0.38

All valence conditions include Unpleasant, Neutral, Harvey, Houston, and Baseline. The Unpleasant and Neutral images both contained humans. The Harvey and Houston images were matched Houston locations before and after Hurricane Harvey. The Baseline condition included an additional filler item

^a^Some tests violated the assumption of sphericity and the degrees of freedom reflect Greenhouse–Geisser correction

^b^*BF*_inc_ refers to the Bayes Factor in favor of including the effect, calculated across matched models

Overall, the results suggest that crisis-related stimuli, specifically images related to Hurricane Harvey, at most yield a weak EAB, if any, in a Houston undergraduate population. This tragedy was a widespread naturally occurring crisis event that was a major stressor to citizens in the Houston area, and its devastating effects could still be seen at the time of data collection in 2021. Still, images of the damage left by Hurricane Harvey did not capture attention to create a strong blink in healthy undergraduate students affected by the stressful event. However, because Experiment 1 used a crisis event that occurred four years prior to data collection, this experiment was examining the *lingering* effects of a tragedy. Thus, Experiment 2 was conducted to see if a *current* stressful event could elicit an EAB—specifically, the COVID-19 pandemic.

## Experiment 2

The COVID pandemic has led to global lockdowns and, at the time of this writing in 2022, nearly 7 million deaths worldwide (World Health Organization, 2020; data updated regularly). Importantly, the COVID pandemic led to a drastic increase in mental health concerns across the general population (Cullen et al., 2020; Pfefferbaum & North, 2020), and young people (ages 18–34) experienced the highest COVID-related mental distress of any age group (Na et al., 2022). In addition, while data from Experiment 1 were collected four years after the tragedy, and thus examined if lingering stress from a past crisis can yield an EAB, data for Experiment 2 were collected between September 18th, 2021 and November 4th, 2021, while the COVID pandemic was still ongoing. This is supported by the fact that, through the duration of data collection, the Harris County (where Houston is located) COVID vaccination rate was only between 50 and 60% (Democrat and Chronicle, 2021; data updated regularly), between 549 and 892 new COVID cases were being reported daily (Harris County Public Health & Houston Health Department, 2022; data updated regularly), and 72% of University of Houston undergraduate psychology courses were still being held virtually. In addition, because the current study used both a past crisis (Hurricane Harvey; Experiment 1) and a current crisis (COVID pandemic; Experiment 2), it can assess the effects of both lingering and current stressors (and whether they are similar or distinct).

Therefore, the COVID pandemic was an ideal stressor to use with the college-aged target sample in the current study and thus, Experiment 2 implemented an EAB paradigm with word stimuli to utilize words related to the COVID pandemic as the stress-related emotional distractors. Experiment 2 sought to determine if COVID-related words result in a similar or greater EAB effect compared to conventional EAB word stimuli (taboo words).

### Method

#### Participants

A total of 40 University of Houston students (31 females, 8 males, 1 non-binary; *M*_age_ = 22.33, SD_age_ = 6.02) participated in Experiment 2 for course credit through the university’s SONA system. One additional participant was excluded from the analyses because they failed to follow instructions, which resulted in 0% accuracy across all conditions. Participants met all of the inclusion criteria outlined in Experiment 1. Informed consent was gathered from all participants under a protocol approved by the University of Houston Institutional Review Board.

#### Design and procedure

**Fig. 3:**
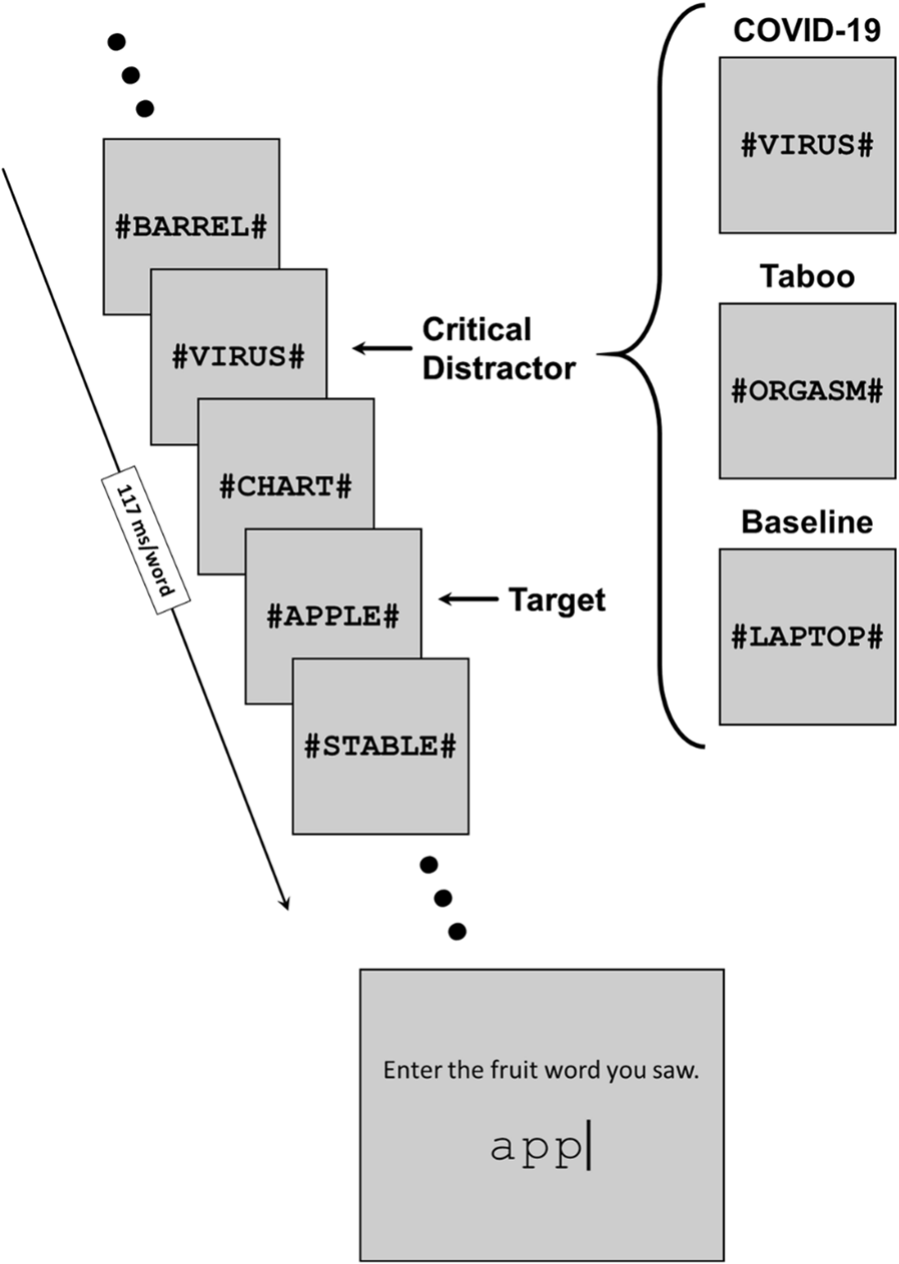
Visual representation of the RSVP task used in Experiment 2. Participants viewed a stream of words presented at a rate of 117 ms per word with a fruit target words and a critical distractor preceding the target. Following each trial, participants entered the fruit word they saw using their keyboard. The right side of the figure shows examples of critical distractors from the COVID, taboo, and neutral valence conditions. The trial stream here depicts a COVID lag 2 trial. For clarity in this illustration, only a single leading and trailing pound sign is displayed; in the actual task, all words were padded to a length of 12 characters (see main text for details)

To test if stress related to COVID yields an EAB, Experiment 2 used an EAB paradigm with RSVP streams of word stimuli, which is common in the EAB literature and shows the same EAB effect as image-based paradigms (Arnell et al., 2007; Huang et al., 2008; Mathewson et al., 2008). Each trial was started by pressing the space key, which initiated a RSVP stream with 2–4 filler items, a critical distractor, 0 (lag 1), 1 (lag 2), 3 (lag 4), or 7 (lag 8) filler items, the target, and 4–12 additional fillers, for a total of 16 RSVP stimuli per stream (Fig. 3). Each word was presented for 117 ms, which is consistent with previous EAB paradigms using word stimuli (Mathewson et al., 2008; Santacroce et al., 2023), and thus one RSVP stream lasted for a total of 1872 ms. The filler stimuli were common neutral words, the targets were defined as fruit words (thus target selection required semantically processing all RSVP items), and the critical distractors could be one of three valence categories: baseline (critical distractor replaced with a neutral filler item), taboo, or COVID (Fig. 3). Following each trial, participants were instructed to type in the fruit words they saw during that stream using their keyboard.

As with Experiment 1, participants first provided consent and filled out a demographics survey on Qualtrics, completed the screen scale calibration program, were given on-screen instructions, carried out practice trials (three in Experiment 2), and then completed the experiment. The practice trials contained only nine words per stream, never included a critical distractor, and had a feedback screen following each trial. The first practice trial was presented at a rate of 517 ms per image, and that rate decreased by 200 ms for each trial until it reached the experiment’s speed of 117 ms per image on the third practice trial.

With the three valence categories (baseline, taboo, or COVID) and four critical distractor to target lags (lags 1, 2, 4, and 8), Experiment 2 had a total of 12 conditions that each had 30 trials for a total of 360 trials. Each session, including consent, demographic collection, setup procedures, training trials, and the experiment trials, lasted approximately 1.5 h.

#### Apparatus

The apparatus in Experiment 2 was identical to that in Experiment 1.

#### Stimuli

Word stimuli were comprised of 120 neutral filler words, 30 taboo distractors, 30 COVID distractors, and 30 fruit target words, all between four and ten letters long. All words were centered on the screen, presented in black Courier New font, were all capitalized, and had a height of approximately 0.64 cm (made uniform across participants’ monitors by the credit card screen scale calibration). In order to correct for large frame-to-frame visual transients due to differing word lengths, each word was padded with pound symbols (#) so that they were all a total of 12 characters long. The fruit words were carefully selected to ensure that they were common enough to be recognized by participants, and thus obscure (e.g., “DRAGONFRUIT” or “LOQUAT”) and ambiguous fruits (e.g., “AVOCADO” or “TOMATO”) were avoided. The filler words were selected to match the lengths of the other words and never contained food words that might interfere with fruit words.

The COVID words were taken from a list of 60 words related to the COVID pandemic (e.g., “VIRUS” or “LOCKDOWN”) and then narrowed down to the 30 best words using a ranking program adapted from html code found in a Tumblr blog post (Vivi, 2018). Rankings were performed by the authors and four additional laboratory members as part of study design. The program presented two words at a time and those who completed the program were to select which word they felt was most related to the COVID pandemic out of each pair of words. The program then took the responses and ranked all of the words from 1 to 60. Word rankings were averaged across laboratory members, and the top 30 words were selected for the experiment. Crucially, the COVID words were rated as being more negative, arousing, and crisis-related than the neutral words by a separate set of participants (*n* = 47, *p*s < 0.001, BF_10_s ≥ 6477.94; see Additional file 1).

Although the initial intention was to use the list of taboo words from a 2008 study by Mathewson et al. (see also Arnell et al., 2007 and Santacroce et al., 2023), some of the fruit and COVID words needed for the current study (e.g., “QUARANTINE” and “BLACKBERRY”) were longer than the maximum eight characters used in that study. Thus, the taboo words were gathered by first selecting 60 words between four and ten characters long that were deemed “not safe for work” (Jones, 2006) and then narrowing them down to the 30 worst words using the same ranking procedure as for COVID words.

#### Data analyses

All analyses were completed in the JASP statistical program (JASP Team, 2018). The main analysis of interest is a 3 (valence: baseline, taboo, COVID) × 4 (lag: 1, 2, 4, 8) within-subjects ANOVA to indicate a difference in the blinks caused by the different valence categories. In addition to the main overall ANOVA, multiple follow-up ANOVAs were conducted to detect possible EAB effects. First, individual one-way ANOVAs were conducted within each valence category to examine a possible blink effect, indicated by a main effect of lag. Second, valence × lag ANOVAs were conducted to compare each of the emotional conditions (taboo and COVID) to the baseline condition. Finally, to directly compare the conventional EAB effect to the crisis-induced EAB, a valence × lag ANOVA was conducted with the taboo and COVID valence conditions. As in Experiment 1, each ANOVA was conducted alongside a matching Bayesian ANOVA, including calculation of BF_inc_ to quantify support for or against including each effect.

### Results and discussion

**Fig. 4:**
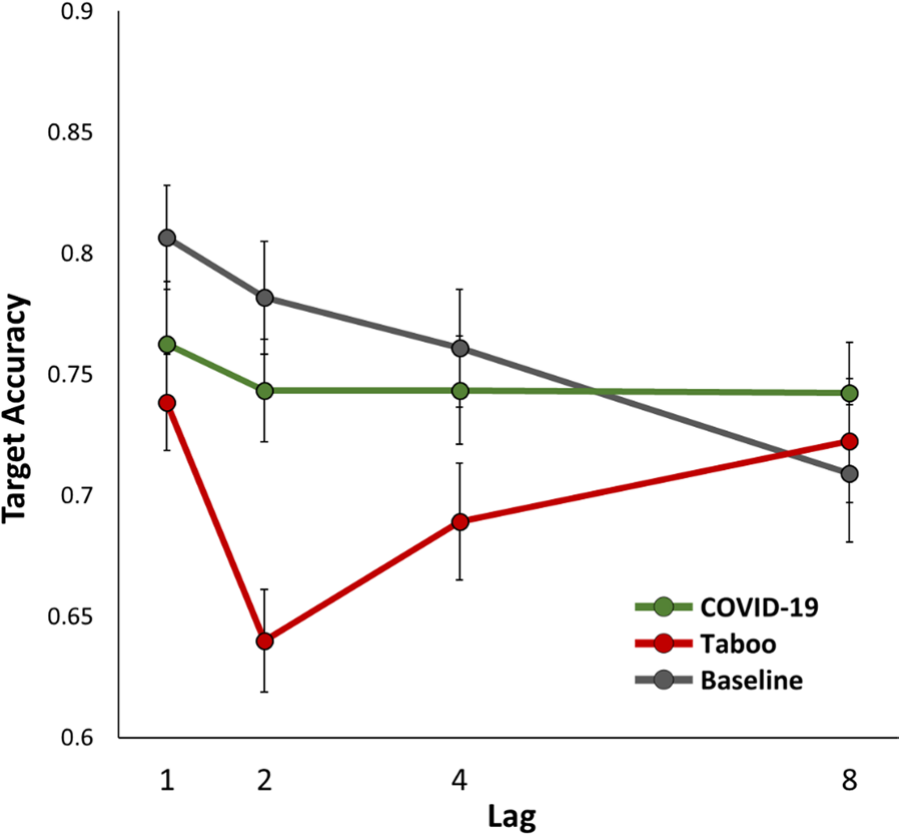
The results of Experiment 2. Error bars represent the standard error of the mean

See Table 2 for full outputs from each ANOVA in Experiment 2. Crucially, the initial 3 (valence: taboo, COVID, baseline) × 4 (lag: 1, 2, 4, 8) within-subjects ANOVA yielded a significant valence × lag interaction (*p* < 0.001, *η*_*p*_^2^ = 0.15, BF_inc_ = 1.43 × 10^4^), indicating a difference in the “blinks” from each valence category (Fig. 4). The results yielded a conventional EAB effect, indicated by a significant valence × lag interaction when the taboo condition was compared to the baseline condition (*p* < 0.001, *η*_*p*_^2^ = 0.22, BF_inc_ = 9.59 × 10^3^) and by a significant simple main effect of lag in the taboo condition (*p* < 0.001, *η*_*p*_^2^ = 0.19, BF_inc_ = 981.48). On the other hand, the stress-induced (COVID) blink was ambiguous—while there was not a simple main effect of lag in the COVID condition (*p* = 0.539, *η*_*p*_^2^ = 0.02, BF_inc_ = 0.08), there was a valence × lag interaction when the COVID condition was compared to the baseline condition (*p* = 0.008, *η*_*p*_^2^ = 0.10, BF_inc_ = 5.43), which was likely driven by the surprising decline in performance with lag in the baseline condition (*p* < 0.001, *η*_*p*_^2^ = 0.20, BF_inc_ = 2.66 × 10^3^). Regardless, the stress-induced COVID blink was much weaker than the conventional EAB caused by taboo words (*p* = 0.002, *η*_*p*_^2^ = 0.12, BF_inc_ = 5.99).

**Table 2:** Results for each analysis of variance ran in Experiment 2

Source	*df*^a^	*F*	*p*	*η*_*p*_^2^	*BF*_inc_^b^
All valence conditions × lag					
Lag	(3, 117)	7.42	< .001	0.16	346.30
Valence	(2, 78)	29.59	< .001	0.43	7.20 × 10^8^
Valence × Lag	(6, 78)	7.12	< .001	0.15	1.43 × 10^4^
Taboo vs. baseline × lag					
Lag	(2.47, 96.44)	7.95	< .001	0.17	218.27
Valence	(1, 39)	43.43	< .001	0.53	2.42 × 10^7^
Valence × Lag	(3, 39)	11.16	< .001	0.22	9.59 × 10^3^
COVID vs. baseline × lag					
Lag	(3, 117)	7.83	< .001	0.17	305.32
Valence	(1, 39)	4.59	.038	0.11	0.69
Valence × Lag	(3, 39)	4.13	.008	0.10	5.43
Taboo vs. COVID × lag					
Lag	(3, 117)	6.32	< .001	0.14	127.80
Valence	(1, 39)	30.536	< .001	0.44	7.59 × 10^4^
Valence × Lag	(3, 39)	5.18	.002	0.12	5.99
Simple main effects of lag					
Taboo	(3, 117)	9.04	< .001	0.19	981.48
COVID	(3, 117)	0.73	.539	0.02	0.08
Baseline	(3, 117)	9.99	< .001	0.20	2.66 × 10^3^

All valence conditions include COVID, Taboo, and Baseline. The Baseline condition included an additional filler word

^a^Some tests violated the assumption of sphericity and the degrees of freedom reflect Greenhouse–Geisser correction

^b^*BF*_inc_ refers to the Bayes Factor in favor of including the effect, calculated across matched models

Experiment 2 examined whether stimuli associated with a contemporaneous crisis event—namely words associated with the COVID-19 pandemic—could elicit an EAB in a healthy undergraduate population. This was in contrast to Experiment 2 that examined the effects of lingering stressors using images related to the damages left by Hurricane Harvey that occurred four years prior to data collection. Still, the COVID-related words yielded, at most, a weak EAB, if any. This supports the results from Experiment 1 and suggests that crisis-related stimuli do not yield a strong EAB in healthy university students, even when related to an ongoing stressful event.

## General discussion

The current study examined whether stimuli related to widespread crises can elicit an equivalent or stronger EAB than conventional emotional stimuli in samples from the general undergraduate population. Given that combat-related stimuli can increase the EAB effect in combat veterans with PTSD (Olatunji et al., 2013) and that stimuli for which stress was artificially elicited in a laboratory setting can lead to stress-induced EABs (Verwoerd et al., 2009), it was possible that similar results might be found with naturally occurring widespread crises in healthy populations. However, through two EAB experiments—one using distractor images related to a past crisis (Hurricane Harvey; Experiment 1) and one using distractor words related to a crisis that was current at the time of data collection (the COVID pandemic; Experiment 2)—the current results suggest that, instead, the stress-induced EABs caused by crisis-related stimuli do not compare to EABs caused by conventional EAB stimuli. In fact, the words related to the COVID pandemic and images related to Hurricane Harvey at most elicited extremely weak EABs, suggesting that stress-related distractors do not capture attention away from current goals in RSVP streams, at least in the general university student population.

### Limitations

The current study has a number of noteworthy limitations. First, the four-year delay between Hurricane Harvey and data collection for Experiment 1 could have dampened the salience of the crisis events and related stimuli. However, since Hurricane Harvey, Houston has experienced two other major flooding events, Tropical Storm Imelda in 2019 and Tropical Storm Beta in 2020, and with its increasing number of minor floods resulting from thunder storms, it is thought to be “America’s rainfall capital” (Erdman, 2021). Thus, the fear of major floods is very prevalent in the city of Houston. Further supporting the ongoing emotional potency, ratings of valence, arousal, and representativeness of the Harvey crisis in a new sample—nearly two years after the Experiment 1 data collection—revealed that the Harvey images were higher in each dimension than the matched Houston images. In addition, Experiment 1 should be understood as a test of the ability of moderately distant, *lingering* stressors to capture attention. Experiment 2, on the other hand, used the COVID pandemic, a crisis that was very much ongoing at the time of data collection and should be understood as a test of the ability of *current* stressors to capture attention. Thus, taken together, the results for Experiments 1 and 2 suggest that neither stimuli related to a lingering stressor nor a current stressor create a strong enough emotional capture to elicit an EAB.

A second limitation is that the crisis-related stimuli may have lost their emotional salience over time as a result of repeated exposure from the news or other media sources, which can dampen participants’ responses to emotional stimuli (Tabibnia et al., 2008). However, the same repeated exposure assertion could be made about the conventional emotional stimuli, and both the Harvey and COVID emotional potency ratings still revealed elevated arousal and valence nearly two years after the EAB data collection. While the Hurricane Harvey images in Experiment 1 may have been overly showcased in the news and on social media in the wake of the tragedy, young college students are also exposed to disturbing visual stimuli in movies or video games, which could be comparable to the conventional disturbing images that yielded a blink. Similarly, while the COVID words in Experiment 2 are seen and heard all over, the same could be said about the conventional taboo words that yielded a blink, given that they are also commonly used by college students in casual settings and have been for longer than words related to COVID.

Third, the participants of the study were young college students (mean ages 21 and 22), and the results may not generalize to other age groups. In the case of Hurricane Harvey, while the event and resulting damage was certainly aversive to the then-teenage participants, the burden of handling the aftermath likely fell mostly on their adult parents or guardians. In the case of the COVID pandemic, young, healthy adults were much less likely to have had a severe case of the disease, and thus may not have experienced as much stress. On the other hand, it has been shown that those exposed to crises earlier in life are more susceptible to lasting psychological effects (Dunn et al., 2017). In the case of COVID, younger individuals experienced more stress as a result of the COVID pandemic than older individuals (Na et al., 2022; World Health Organization, 2022a, 2022b). In addition, young college-aged individuals were forced to miss many milestones, such as high school prom and graduation, living on campus during their first years of college, taking in-person classes, and having general social interactions. These missed milestones and the associated isolation likely play a large role in their mental health. Notably, University of Houston students are a population that may be less insulated from the effects of crisis compared to university students nationally, given the student body’s ethnic diversity, family education level, and lower income status (CollegeSimply, 2023; University of Houston, 2022).

Fourth, the goal of the present study was to examine effects in a subset of the general population (college students), rather than in people who had been previously diagnosed with PTSD or screened by other clinical measures. In order to meet this goal, the current study explicitly did not take into account the individual impacts of the events on participants beyond verifying their exposure (i.e., that they resided in Houston during Harvey for Experiment 1). Thus, it may be that some participants were more affected by the events than others. However, with this in mind, additional analyses failed to observe increased variance in the crisis-induced blinks compared to the traditional EAB, which is inconsistent with a large impact of this limitation.^1^

Finally, the present study examined two categories of crises that led to only weak EABs, but more severe events (e.g., exposure to a mass shooting) may evoke larger EABs, while more mild exposures (e.g., events that might be considered merely annoying) might evoke no EAB at all. Regardless, it seems unlikely that any but the most severe of traumatic events—events that are likely to lead to widespread consequent PTSD—can evoke a larger EAB than that found with conventional unpleasant (Experiment 1) or taboo (Experiment 2) stimuli.

### Implications for future research

The current study expanded on previous research that examined the effects of stimuli related to stressful events on the EAB. Specifically, previous studies have mainly focused on clinical populations, such as exposing combat veterans with PTSD to combat-related images (Olatunji et al., 2013), or have relied on artificially induced stressful stimuli in the laboratory (Verwoerd et al., 2009). The current study instead used stimuli related to widespread crises (Hurricane Harvey and the COVID-19 pandemic) in a healthy undergraduate population to see if they could elicit an EAB, which would indicate strong stimulus-driven temporal attentional capture by stress-related stimuli. The results thus shed light on how both lingering and ongoing crises influence the control of selective attention in a subclinical population, which has often been neglected in attentional capture literature. The current results suggest that these stimuli do not take priority over current goals, at least in the EAB task.

On the other hand, although the EAB task could be diagnostic of the persisting impact of emotionally salient distractors (Onie & Most, 2017), crisis-related stimuli have the potential to impact other aspects of attention affected by emotion in a general population. For example, consistent with some studies using typical emotional stimuli, these crisis-related stimuli could narrow the focus of attention in contextual cueing tasks or Navon tasks (Fenske & Eastwood, 2003; Fredrickson, 2004; Gable & Harmon-Jones, 2010; Kunar et al., 2014, 2014), or capture spatial attention in visual search or dot probe tasks (Fabio & Caprì, 2019; Onie & Most, 2021; Zsido et al., 2020). These other aspects of emotional impact on attention are not tested here, and future research could use similar widespread crises in non-clinical populations to test these other measures of attention.

### Conclusion

The results of the current study show that stimuli related to widespread crises do not result in a strong EAB effect in the general public, either several years after the crisis or while the crisis is ongoing. Future studies should follow up on the present approach by directly comparing samples who do and do not meet clinical standards for PTSD diagnosis in response to the identical crisis events. More broadly, the present study represents a blueprint for how future studies could examine the magnitude of lasting emotional impact from a crisis or other negative event. More directly, the present results are important in that they address whether findings from extreme cases of emotional salience in clinical populations (e.g., combat images in combat-veteran PTSD patients) generalize to long-term effects from the broader population’s experience of disasters and other stressful events.

### Supplementary Information

**Additional file 1. **Methods, results, and discussion for the ratings of stimulus valence, arousal, and crisis-relatedness reported in the Method sections for each main experiment.

## Data Availability

All data, experimental code, and permitted experimental stimuli have been made available on Open Science Framework (https://osf.io/skp9q/?view_only=6dc5e12228a34a5d849f85679b1e3cf2). The available stimuli do not include the emotional images from Experiment 1, which were IAPS images and are not to be publicly shared. However, the numerical image names, as well as valence and arousal ratings, from the IAPS database are available for reference; this information allows anyone with access to the IAPS image set to reconstruct the stimuli used in these experiments.

## Footnotes

1 To see if variability in participants’ experiences with the crisis events used in this study (Hurricane Harvey and the COVID pandemic) explained the weakness of the crisis-induced EAB, F tests were conducted to compare task performance variance in the crisis conditions to those in the traditional blink condition (unpleasant or taboo). If there was increased variability at the lag with the greatest blink—lag 2—during the crisis-related conditions compared to the traditional conditions, it could suggest that the heterogeneity of the participants’ varying experiences played a role in the current study’s results. However, this was not the case—the variances did not significantly differ between the two conditions both in Experiment 1, *F*(1, 39) = 1.44, *p* = .131, and Experiment 2, *F*(1, 39) = 1.01, *p* = .485.
