# Supplementary material for: Crisis-related stimuli do not increase the emotional attentional blink in a general university student population
Source: Cogn Res Princ Implic. 2024 Jan 8;9:3. doi: 10.1186/s41235-023-00525-7 (PMC10774501; doi:10.1186/s41235-023-00525-7)
Supplement: Supplementary file 1 — Additional file 1. Methods, results, and discussion for the ratings of stimulus valence, arousal, and crisis-relatedness reported in the Method sections for each main experiment. [file 41235_2023_525_MOESM1_ESM.docx]

# Supplement

## Method

### Participants

A total of 47 University of Houston students (40 females, 6 males, 1 nonbinary; M_age_ = 22.70, SD_age_ = 5.62) participated in this experiment for course credit through the university’s SONA system. Participants met all of the inclusion criteria outlined in the main experiments. Thus, they were from the same participant pool as in the main experiments, but did not participate in Experiment 1 or 2. Of note, this experiment was conducted two years after data collection from the main study (May – August 2023). Informed consent was gathered from all participants under a protocol approved by the University of Houston Institutional Review Board.

### Design

The purpose of this study was to rule out the alternative account that small or absent blinks evoked by crisis-related stimuli could stem from a lack of potency of those stimuli, presumably due to the time elapsed since Hurricane Harvey (Experiment 1) or the onset of the COVID-19 pandemic (Experiment 2). The delay from collection of the main data to the stimulus ratings described here works in favor of being able to rule out this alternative, because it would be expected that this additional delay could only erode the potency of these crisis-related stimuli further. Thus, the results of the present ratings should be taken as minimum estimates on the valence, arousal, and crisis-relatedness of the stimuli. Participants rated relevant stimuli on all three dimensions in a single online session.

### Procedure

In order to test if the crisis-related stimuli used in the current study (Hurricane Harvey images and COVID words) were sufficiently salient and crisis-related, participants rated the valence, arousal, and crisis relatedness of the stimuli and their neutral counterparts. If the crisis-related stimuli were rated as more crisis-related, it would indicate that the stimuli used in the main experiments sufficiently tested the effects of a stress-induced EAB from crisis-related stimuli. On the other hand, if there were no difference between the crisis-related stimuli and their neutral control stimuli, it would suggest that the current study failed to observe the EAB effect simply because of ineffective stimulus selection. Similar logic can be applied for valence and arousal.

The apparatus in the rating experiment was identical to Experiments 1 and 2 of the main text. Participants first provided consent and filled out a demographics survey on Qualtrics, were given on-screen instructions, and then completed the stimuli ratings. The rating task consisted of two blocks of trials presented in a random order: one to rate the Hurricane Harvey and Houston images used in Experiment 1 and one to rate the COVID and neutral words used in Experiment 2. Each trial presented a stimulus (image or word, depending on block) centered on the screen with three 9-point Likert-like scales (Likert, 1932) presented below, and participants were instructed to use their mouse to rate each stimulus’s valence (1 = very negative, 9 = very positive), arousal (1 = not at all arousing, 9 = very arousing), and crisis-relatedness (1 = not at all relating to the crisis, 9 = very related to the crisis). In the Harvey block, “crisis-relatedness” was defined as how related each image is to the damages caused by Hurricane Harvey. In the COVID block, “crisis-relatedness” was defined as how related each word is to the COVID-19 pandemic. With a total of 96 images and 150 words to rate, there were a total of 246 trials.

### Stimuli

The stimuli in the rating experiment consisted of the crisis-related stimuli and their neutral counterparts that were identical to those used in Experiments 1 and 2. Therefore, the Hurricane Harvey block contained the full set of images of Houston in the wake of Hurricane Harvey (crisis-related “Harvey” condition) and before Hurricane Harvey (neutral “Houston” controls). Similarly, the COVID block contained the 30 COVID-related words and the 120 neutral words. Of note, one of the Harvey images in the Hurricane Harvey block was accidentally replaced with a duplicate Houston image, and thus the ratings only characterize 47 of the 48 Harvey images.

# Results and Discussion

All analyses were completed in the JASP statistical program (JASP Team, 2018). Paired t-tests and accompanying Bayesian paired t-tests were conducted to compare the valence, arousal, and crisis-relatedness ratings in the crisis conditions to their neutral control conditions. For these analyses, ratings were averaged across exemplars within category for each participant in order to match the main experiments, in which conditions were based on stimulus category, not exemplar. The results showed that the crisis conditions (both the Harvey images and the COVID words) were significantly more negative, arousing, and crisis-related than their neutral control stimuli. This suggests that the stimuli used in the current study were sufficient to examine the effects of crisis-related stimuli on the EAB, and that the lack of an EAB was not because participants did not find the stimuli negative, arousing, or even crisis-related. See Table S1 for the paired t-test results and OSF for the full data set.

| Table S1 | | | | | | | | | |
| --- | --- | --- | --- | --- | --- | --- | --- | --- | --- |
| *Paired t-test Results Comparing Valence, Arousal, and Crisis-relatedness Ratings for the Crisis-related Stimulus Category and Neutral Control Stimulus Category* | | | | | | | | | |
| Rating | | Crisis-related | | Neutral | | *t*(46) | *p* | Cohen’s *d* | BF_10_ |
|  | | *M* | *SD* | *M* | *SD* |  |  |  |  |
| Hurricane Harvey and Neutral Images (Experiment 1) | | | | | | | | | |
|  | Valence | 2.40 | 0.92 | 5.51 | 0.77 | -16.29 | <.001 | -2.38 | 4.57×10^17^ |
|  | Arousal | 4.72 | 2.35 | 3.01 | 1.63 | 3.97 | <.001 | 0.58 | 100.37 |
|  | Crisis-relatedness | 7.55 | 2.21 | 2.21 | 0.99 | 21.24 | <.001 | 3.10 | 1.63×10^22^ |
| COVID-19 and Neutral Words (Experiment 2) | | | | | | | | | |
|  | Valence | 3.36 | 0.94 | 5.19 | 0.40 | -12.37 | <.001 | -1.80 | 2.01×10^13^ |
|  | Arousal | 5.13 | 1.90 | 3.39 | 1.61 | 5.35 | <.001 | 0.78 | 6,477.94 |
|  | Crisis-relatedness | 7.89 | 0.88 | 2.98 | 1.26 | 21.93 | <.001 | 3.20 | 6.10×10^22^ |
| *Note.* In the Hurricane Harvey block, participants rated two image categories used in Experiment 1: the crisis-related images of Houston after Hurricane Harvey and the matched neutral control images of Houston. In the COVID-19 block, participants rated two word categories used in Experiment 2: the crisis-related words related to the COVID-19 pandemic and neutral words. | | | | | | | | | |
